# Magneto radiative and heat convective flow boundary layer in Maxwell fluid across a porous inclined vertical plate

**DOI:** 10.1038/s41598-023-33477-5

**Published:** 2023-04-17

**Authors:** K. Sudarmozhi, D. Iranian, Ilyas Khan, Amnah S. Al-johani, Sayed M. Eldin

**Affiliations:** 1 Department of Mathematics, Saveetha School of Engineering, SIMATS, Chennai, Tamil Nadu India; 2 Department of Mathematics, Saveetha School of Engineering, SIMATS, Chennai, Tamil Nadu India; 3 Department of Mathematics, College of Science Al-Zulfi, Majmaah University, Al-Majmaah, 11952 Saudi Arabia; 4 Mathematics Department, Faculty of Science, University of Tabuk, Tabuk, Saudi Arabia; 5 Faculty of Engineering, Center of Research, Future University in Egypt, New Cairo, 11835 Egypt

**Keywords:** Energy science and technology, Engineering

## Abstract

Heat transport in a 2D steady radiative boundary layer with Maxwell fluid flow and the influence of heat generation and MHD has been studied across a porous inclined vertical plate. Through similarity transformation, the mathematical modelling is converted to ODEs, and the built-in solver Bvp4c via MATLAB is used to solve. The linear movement of an inclined porous plate introduced the flow. The MHD (*M*), Prandtl number (*Pr*), radiation (*Rd*), Rayleigh number (*Ra*), local Nusselt number (*Nu*_*x*_), angle of inclination (*γ*), and material relaxation time (*β*) have a considerable impact on the flow field as a result. The local Nusselt numbers and the skin friction coefficient are also given as numbers. The validation with the numerical solution is presented. The results are shown, and a thorough physical analysis has been done. The temperature of the fluid rises due to the greater electric field, increasing the heat transfer on the inclined plate. However, skin friction increases dramatically as the heat radiation parameter rises. The critical findings of this study are that the temperature profile increases and the velocity profile lower as the inclination angle increases. The Maxwell fluid parameter raises the velocity profile as well.

## Introduction

Free convection is the principal mechanism in many of the heat transport phenomena that occur around us. Natural convection plays a significant role in cooling electrical equipment, such as heat transfer from refrigeration coils, power transistors, and power transmission lines, and heat transfer from the bodies of animals and humans. Free convection cooling is regarded as the most effective and efficient cooling process. Free convection occurs due to a temperature differential between the body and the surrounding air, which creates a difference in the density of the air, resulting in a density difference and buoyant force, which is the primary cause of free convection currents. Pandya and Quraishi^1^ investigated the result of heat absorption or generation flow on MHD through an inclined semi-infinite plate over a porous material with varying thicknesses. Rajput and Gaurav^2^ explored the Soret result on non-time dependent MHD flow by using an impulsively begun inclined wavering plate with the effect of variable temperature. In the occurrence of nanoparticles, Arifuzzaman et al.^3^ analyzed the MHD Maxwell fluid flow on a perpendicular porous plate along with radiation absorption, chemical reaction and heat generation. Hosseinzadeh et al.^4^ thought that the possessions of nonlinear radiation and reactive chemical flow on the Maxwell fluid across a porous media equipped with a convectively heated plate are investigated. Mishra and Samantara^5^ investigated the impacts of electrification and radiation on a non-time-dependent two-phase flow across an inclined permeable stretching sheet. Khan et al.^6^ investigated how thermal radiation and chemical reactivity affect non-Newtonian fluids as they pass over a perpendicularly stretched permeable plate with uniform suction. The influence over free convection, heat transmission and mass transfer was explored by Islam et al.^7^ about an inclined plate embedded in a permeable media. Huang^8^ discussed the outcome of thermophoresis over mass transfer from free convective flow transversely to a perpendicular permeable medium with the effect of variable wall heat fluxes. Rahman et al.^9^ studied this topic in their study titled heat transfer of a heat production MHD fluid flow through a vertically permeable flat plate in a rotating system.

The permeable medium plays a significant part in managing momentum and heat transfer over a boundary layer flow of various fluids. Given this fact, many writers have investigated porous matrices’ influence on non-Newtonian and Newtonian fluids. Many writers using non-Newtonian fluids examined the effect of permeability. As a result of this, there has been a considerable uptick in interest in heat transfer issues that involve the usage of non-Newtonian fluids. Understanding the physics that underlies the flow of these fluids can take an immediate impact on many different processes. These processes include coating, turbulent shear flow, ink-jet pointing, micro fluids, polymer processing, colloidal suspensions, geological flows in the earth’s mantle, liquid crystals, animal blood, and additive breaks. Non-Newtonian flow often demonstrates properties such as shear-thinning, shear thickness, and viscoelasticity. As a direct significance of this, there is a substantial body of written work on analytical and numerical solutions and a significant interest in investigating non-Newtonian flows. Tahir et al.^10^ examined the impact of non-integer wall slip derivatives with the effect of heat transfer flow on Maxwell fluid across an oscillating perpendicular plate using a novel concept of fractional Caputo-Fabrizio derivatives. Riaz et al.^11^ discussed the solutions of ramping wall velocity, temperature and concentration for unsteady convection of an MHD Maxwell fluid based on special functions. Nadeem et al.^12^ observed the chemically reactive species in a Maxwell fluid flow. Riaz et al.^13^ looked into the possibility of developing new particular solutions for a generalized Maxwell Fluid flow. The effect of ramped wall velocity and temperature on unsteady MHD convective Maxwell Fluid flow was observed by Anwar et al.^14^. Aman et al.^15^ discussed the MHD effect on a nano-Maxwell fluid moving through a plate with a porous media containing cobalt nanoparticles. The Fluid was moving through the plate. MHD Powell–Eyring nanofluid motion across a perpendicular plate with the influence of convective surface conditions with the result of Dufour–Soret using Lie group analysis done by Ogunseye et al.^16^. Alagumalai et al.^17^ explored how to improve the efficiency of nano-enhanced heat exchanger/energy storage systems and a proper action plan to overcome the barriers. Tayebi et al.^18^ investigated the thermo-free convection and entropy formation of an Al2O3-H2O nanofluid surrounded by two circular cylinders in the occurrence of magnetic fields. Chamkha et al.^19^ used the control-volume-based CVFEM to computationally study the free convection of an MHD nanofluid in an enclosure under the influence of thermal radiation. Dogonchi et al.^20^ investigated the effects of thermal radiation, viscous dissipation, and Joule heating on squeezing flow current and the heat transfer mechanism in parallel discs during a suction/blowing process for an MHD nanofluid flow. Eshaghi et al.^21^ examined the DDNC of a hybrid Cu–Al2O3–water nano liquid inside an H-shaped hollow with a top wall baffle. Dogonchi et al.^22^ investigated the heat transmission and entropy generation of a magnetic Fe3O4–H2O nano liquid inside a porous cage using two square cylinders. Afshar et al.^23^ investigated the dissection of entropy production for natural convection in a permeable wavy enclosure filled with NEPCMs and subjected to a volumetric heat source/sink. Pasha et al.^24^ investigated the effect of exothermic reactions on the thermal behaviour of nano-encapsulated phase change materials (NEPCMs) particles suspended in a complicated adiabatic enclosure comprised of two differently heated circular cylinders. Natural convection heat transfer in a porous prismatic enclosure with two movable hot baffles was examined by Shao et al.^25^. Pasha et al.^26^ studied heat exchange, natural convective flow, and entropy-generating features in a hexagonal-shaped region controlled by an inclined magnetic field and filled with a non-Newtonian shear-thickening fluid charged with Alumina nanoparticles. Mondal et al.^27^ created CuO–H2O nanofluids in a 2D circular geometry with a rhombus-shaped barrier that keeps two neighbouring high walls at the same temperature. Zidan et al.^28^ provided a modelling approach for predicting thermal free convection inside a permeable trapezium-shaped zone with adiabatic side-wall baffles. Seyyedi et al.^29^ presented a Second law study of magneto-free convection in a wavy-hexagonal permeable enclosure filled with nanofluid. Nuwairan et al.^30^ studied the flow of a Maxwell fluid with heat transfer via a permeable medium using thermophoresis particle deposition and Soret–Dufour impacts. Saqib et al.^31^ investigated heat transfer in Maxwell Fluid MHD flow using a fractional Cattaneo–Friedrich model. Hayat et al.^32^ observed Maxwell Fluid steady flow with convective boundary conditions. Khan et al.^33^ explored the mathematical study of double-diffusive in a Maxwell fluid. Muhammad et al.^34^ explored the combined impacts of heat and mass transfer on Maxwell fluid MHD free convection flow with variable temperature and concentration. Gangadhar et al.^35^ used the spectrum relaxation method to describe numerical computation for steady boundary layer flow of Maxwell fluid on a stretching surface embedded in a permeable medium with viscous dissipation. Zhao et al.^36^ explored fractional MHD Maxwell Fluid convection heat and mass transfer in a permeable medium with Soret and Dufour impacts. Ramzan et al.^37^ studied Maxwell fluid slippage flow on an inclined perpendicular plate with generalized heat and mass transfer. MHD viscoelastic fluid flows past an infinite vertical plate in the presence of radiation, and the chemical reaction was investigated by Raju et al.^38^. Natural convection simulation of Prabhakar-like fractional Maxwell fluid moving on an inclined plane with generalized thermal flux was investigated by Khan et al.^39^. Bai et al.^40^ studied unsteady inclined stagnation point flow and Maxwell fluid thermal transmission on a stretched/contracted plate with a changed pressure field. Ramzan et al.^41^ studied the effect of diffusion-thermo on Maxwell fluid MHD flow with heat and mass transfer. Much research has recently been conducted on hydromagnetic fluxes and heat transmission in permeable media. The rationale stems from their various technical procedures, which include casting, fusion control, liquid metal filtering, and nuclear reactor cooling. In all the above articles, authors studied heat transfer effects in various geometry using various fluids but exactly not in Maxwell fluid over a porous inclined vertical plate.

As per the literature review, we can understand that no one has investigated heat transfer flow in Maxwell fluid over an inclined porous plate with the combination of the parameter MHD, radiation and heat generation. So, in this investigation, we seek to clarify the effects of an inclined perpendicular porous plate on heat generation/absorption, radiation, and MHD. Framed PDEs are transformed into ODEs using similarity transformation. Then these equations are solved by the RK45 method with the help of inbuilt software Bvp4c with the shooting technique through MATLAB. The power of different physical parameters on temperature and velocity field converse with the aid of tables and pictures.

On the other hand, tables are used to discuss critical physical parameters like the Maxwell Fluid parameter, thermal radiation, the magnetic field, the Rayleigh number, and the Prandtl number. We have validated with existing work and got an excellent agreement. This study aims to find the flow field for the inclination angle and Maxwell fluid parameter. Many disciplines use heat transfer technologies and thermal management of electronic devices, including automotive engineering and systems, climate control, insulation, chemical engineering, materials processing, and power station engineering.

## Mathematical formulation

**Figure 1:**
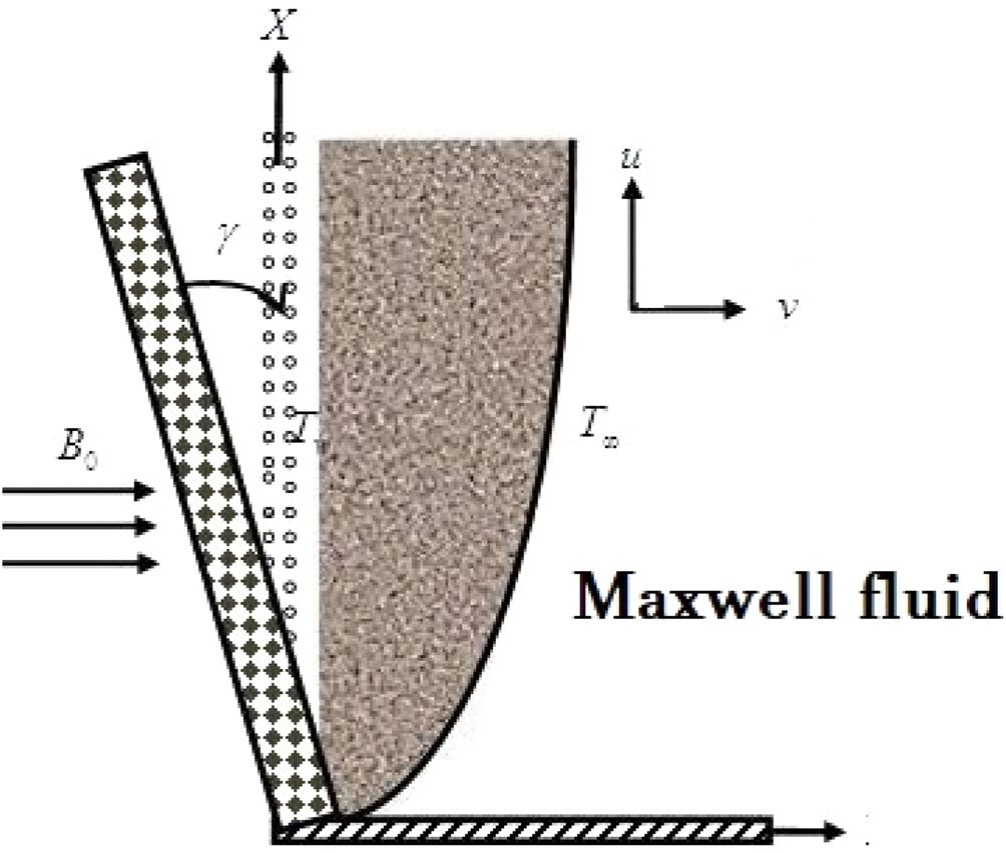
Physical model of the problem (Alam et al.^42^).

In this research, MHD flow plates with an inclination angle have been considered. The plate’s *x*-axis and *y* normal are taken. The governing equations of non-Newtonian fluids were considerably more intricate and nonlinear than those of Newtonian fluids. Constant two-dimensional laminar flow is considered because of its intricacy. The plate’s leading edge restricts the streamwise (*x*) and average (*y*) dimensions. *u* and *v* indicate the velocity mechanisms perpendicular to the flow and vertical to the stream direction. A transverse magnetic field *B*_0_ of constant strength is considered on the flow. Both the induced magnetic field and viscous dissipation have been considered. Initially, the plate and the fluid are both at the same temperature, T, because it is preserved at a temperature *T*_*w*_ that is more complex than the constant temperature *T* of the surrounding fluid (Fig. 1).

The convective flow of an incompressible steady Maxwell fluid subject to the Boussinesq approximation can be characterized by the resulting conservation equations within the framework of the assumptions as mentioned earlier by including the Maxwell parameter from Aydın and Kaya^43^.

1 $$\frac{\partial u}{{\partial x}} + \frac{\partial v}{{\partial y}} = 0.$$

Porous media is finding increasing use in businesses and modern technology. Knowledge of convection in porous media, in particular, is essential in constructing the necessary equipment and better comprehending the phenomenon.

2 $$\begin{aligned} u\frac{\partial u}{{\partial x}} + v\frac{\partial u}{{\partial y}} = & \upsilon \left( {\frac{{\partial^{2} u}}{{\partial y^{2} }}} \right) - \frac{{\sigma B_{0}^{2} }}{\rho }u \\ & - \frac{\upsilon }{k}u\,\, + g\left( {\beta_{T} \left( {T - T_{\infty } } \right)} \right)\cos \gamma - \lambda \left( {u^{2} \frac{{\partial^{2} u}}{{\partial x^{2} }} + v^{2} \frac{{\partial^{2} u}}{{\partial y^{2} }} + 2uv\frac{{\partial^{2} u}}{\partial x\partial y}} \right). \\ \end{aligned}$$

    Thermal radiation is critical in processes with high operating temperatures. Nuclear power plants, propulsion and gas turbines ion equipment for aircraft, missiles, satellites, and space vehicles are only examples of engineering applications with substantial radiative effects.

3 $$\left( {u\frac{\partial T}{{\partial x}} + v\frac{\partial T}{{\partial y}}} \right)\, = \frac{k}{{\rho C_{p} }}\,\frac{{\partial^{2} T}}{{\partial y^{2} }} + \frac{1}{{\rho C_{p} }}\frac{{16\sigma^{*} T_{\infty }^{3} }}{{3k^{*} }}\frac{{\partial^{2} T}}{{\partial y^{2} }} + Q(T - T_{\infty } ).$$

The boundary conditions of a perpendicular plate are as given here

4 $$\begin{gathered} u = 0,\,\,v = 0,\,\,T = T_{w} \,\,at\,y = 0 \hfill \\ u,v \to 0,\,\,\,\,T \to T_{\infty } \,\,\,\,as\,\,\,\,y = \infty . \hfill \\ \end{gathered}$$

Similarity variables and dimensionless quantities are as follows.

5 $$\begin{gathered} \eta = \frac{y}{x}Ra_{x}^{0.25} ,\;{\text{here}}\;Ra_{x} = \frac{{g\beta \left( {T_{w} - T_{\infty } } \right)x^{3} }}{\alpha \upsilon }, \hfill \\ f\left( \eta \right) = \frac{\psi }{{\alpha Ra_{x}^{0.25} }},\,\,\theta \left( \eta \right) = \frac{{T - T_{\infty } }}{{T_{w} - T_{\infty } }}. \hfill \\ \end{gathered}$$

The flow properties often indicated by the stream function $\psi$ are listed below.

6 $$u = \frac{\partial \psi }{{\partial y}},\,\,\,v = - \frac{\partial \psi }{{\partial x}}.$$

By making use of the above quantities, the PDSEs are transformed into ODEs, as given here

7 $$\begin{aligned} f^{\prime\prime\prime}\left( {4\Pr - \frac{9}{4}\beta \sqrt {Ra_{x} } f^{^{\prime}2} } \right) - & \beta \sqrt {Ra_{x} } \left( {\frac{7}{4}\eta f^{^{\prime}2} f^{\prime\prime} - f^{^{\prime}3} - 1.5f\,f^{\prime}f^{\prime\prime}} \right) \\ - & \;4M\sqrt {\Pr } f^{\prime}\, - 4\frac{\Pr }{{\sqrt {Ra_{x} } }}k_{1} f^{\prime} + 3ff^{\prime\prime} \\ - & \;2f^{^{\prime}2} + 4\Pr \theta \cos \gamma = 0, \\ \end{aligned}$$

8 $$\left( {1 + \frac{4}{3}Rd} \right)\theta^{\prime\prime} + \frac{3}{4}f\theta^{\prime} + Q^{ * } \theta \, = 0.$$

The following is the proper equivalent non-dimensional border conditions

9 $$\left. \begin{gathered} f\left( 0 \right) = 0,\,\,\,\,\,f^{\prime}\left( 0 \right) = 0,\,\,\,\,\,\,\theta \left( 0 \right) = 1 \hfill \\ f^{\prime}\left( \infty \right) = 0,\,\,\,\,\,\,\theta \left( \infty \right) = 0 \hfill \\ \end{gathered} \right\},$$

$${\text{Magnetic field parameter}}\;M = \frac{{\sigma B_{0}^{2} x^{1/2} }}{{\rho \sqrt {g\beta \left( {T_{w} - T_{\infty } } \right)} }},$$

$${\text{Radiation parameter}}\;Rd = \,\,\frac{{4\sigma T_{\infty }^{3} }}{k\delta },$$

$${\text{Prandtl number}}\;\Pr = \frac{\nu }{\alpha },$$

$${\text{Thermal diffusivity }} \alpha = \frac{k}{{\left( {\rho c_{p} } \right)}}.$$

Given the velocity field, one may calculate the skin—friction at the plate, which is given in non-dimensional form by

$Cf_{x} = \frac{{\tau_{w} }}{\rho }\,\,{\text{where}}\,\,\tau_{w} = \,\mu \left( {\frac{\partial u}{{\partial y}}} \right)\,\,$ is wall shear stress.

Understanding the temperature field allows one to calculate the rate of heat transfer coefficient, which is expressed in terms of the Nusselt number in non-dimensional form.

$Nu_{x} \, = \frac{{xq_{w} }}{{k\left( {T_{w} - T_{\infty } } \right)}}\,\,,\,\,q_{w} = - k\frac{\partial T}{{\partial y}} + q_{r}$, where *q*_*w*_ is the heat flux.

## Result and discussion

Furthermore, we have yet to be aware of any published research on the combined effect of heat generation and thermal radiation on magnetohydrodynamic natural convection over an inclined plate embedded in a permeable medium. As a result, one of the objectives of this investigation is to incorporate a numerical computation of the heat transfer enhancements generated by magnetohydrodynamic free convection across an inclined plate. Throughout the conversation we consider* M* = 0.5, *Rd* = 0.5, *Pr* = 5, *Ra* = 0.5, *γ* = *pi*/6, *β* = 0.1, Q* = 0.01. For various physical parameters, the heat generation, *M*, and radiation parameters, the numerical solutions of the temperature, velocity, *Nu* and skin friction were computed and analyzed graphically using MATLAB (RK45). The current study was contrasted with the closest solution found in the literature to determine the exactness of the numerical results. The below table (Table 1) shows the validation of the present work with existing work. Valuation results of Nusselt number *Nu* = *Nu/Ra*_*x*_^1/4^ for the regular fluid with *Nr* = *Nb* = *Nt* = 10.

**Table 1:** Comparison of present results with published work^44^.

*Pr*	Kuznetsov and nield^44^	Present
10	0.465	0.462
1	0.401	0.403
100	0.490	0.482

## Graphical results

**Figure 2:**
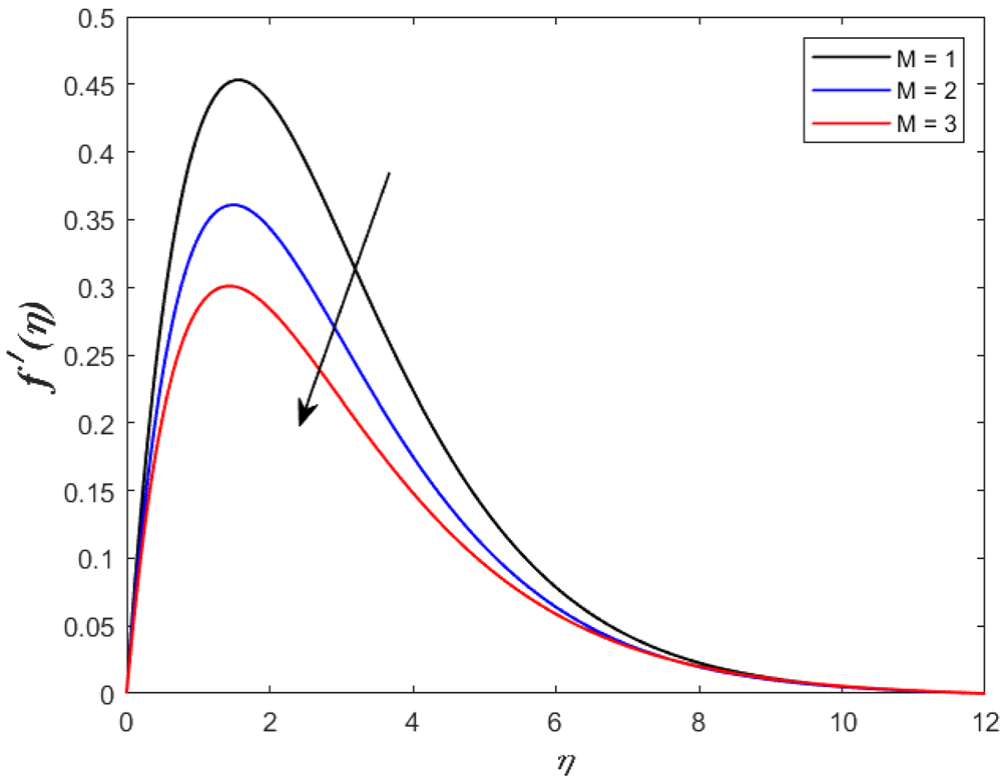
Influence of* M* on the velocity field.

**Figure 3:**
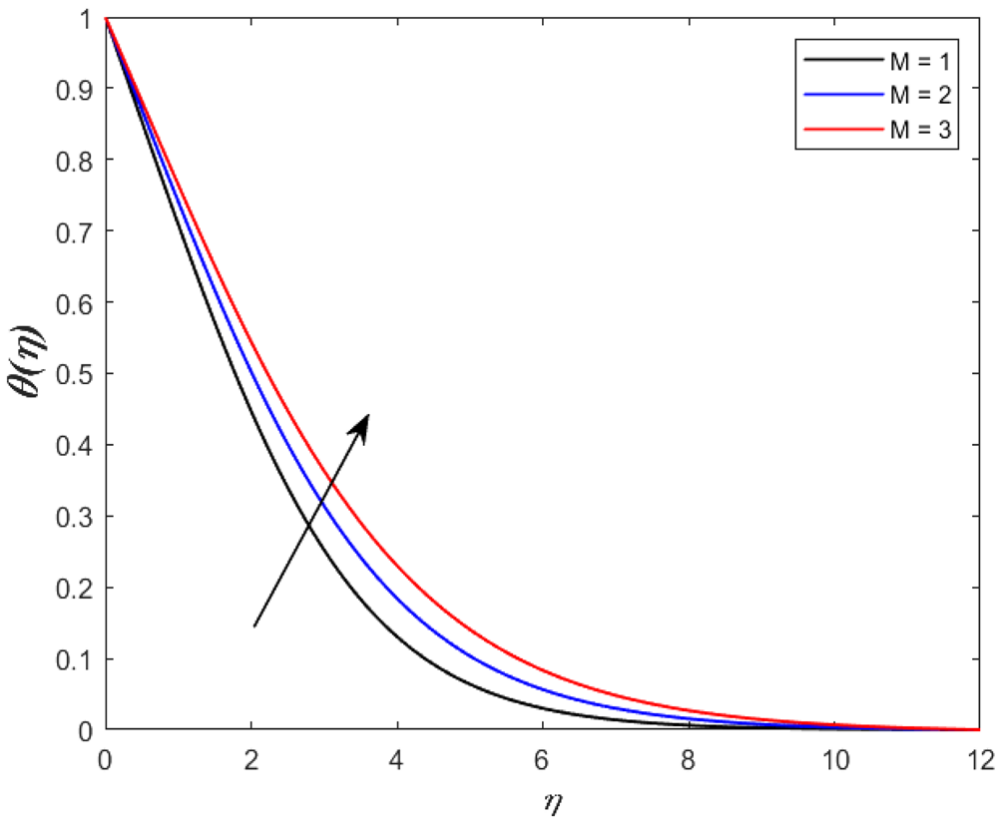
Influence of* M* on the temperature profile.

**Figure 4:**
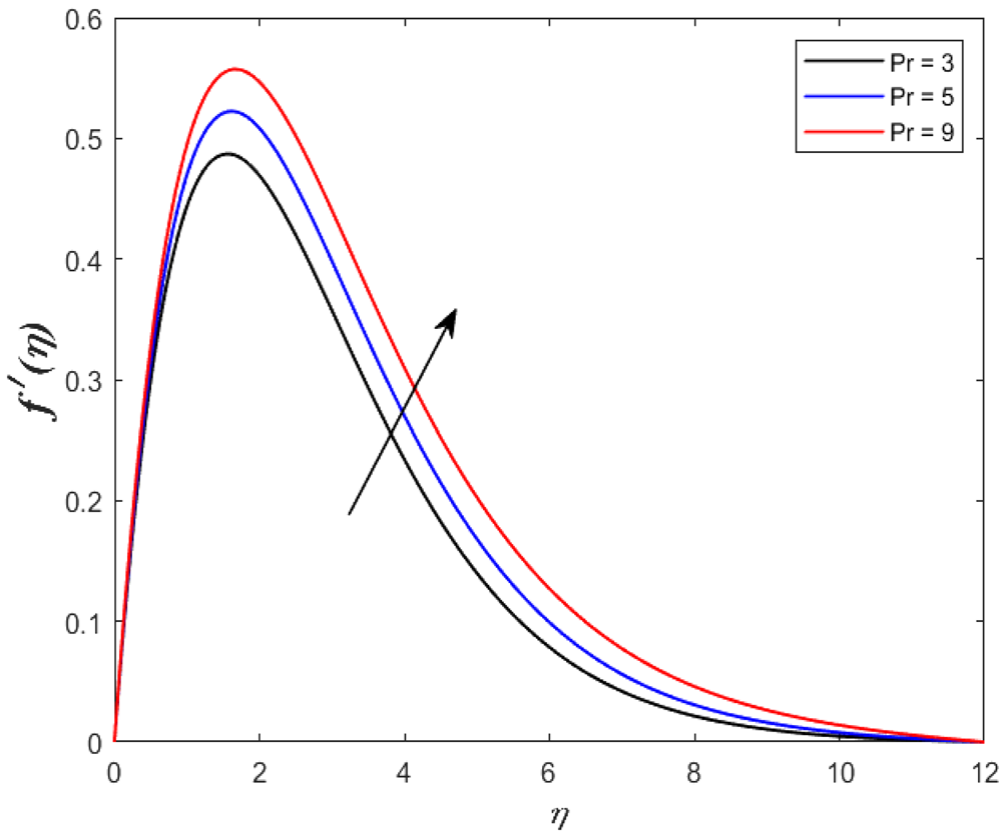
Effect of *Pr* on the velocity outline.

**Figure 5:**
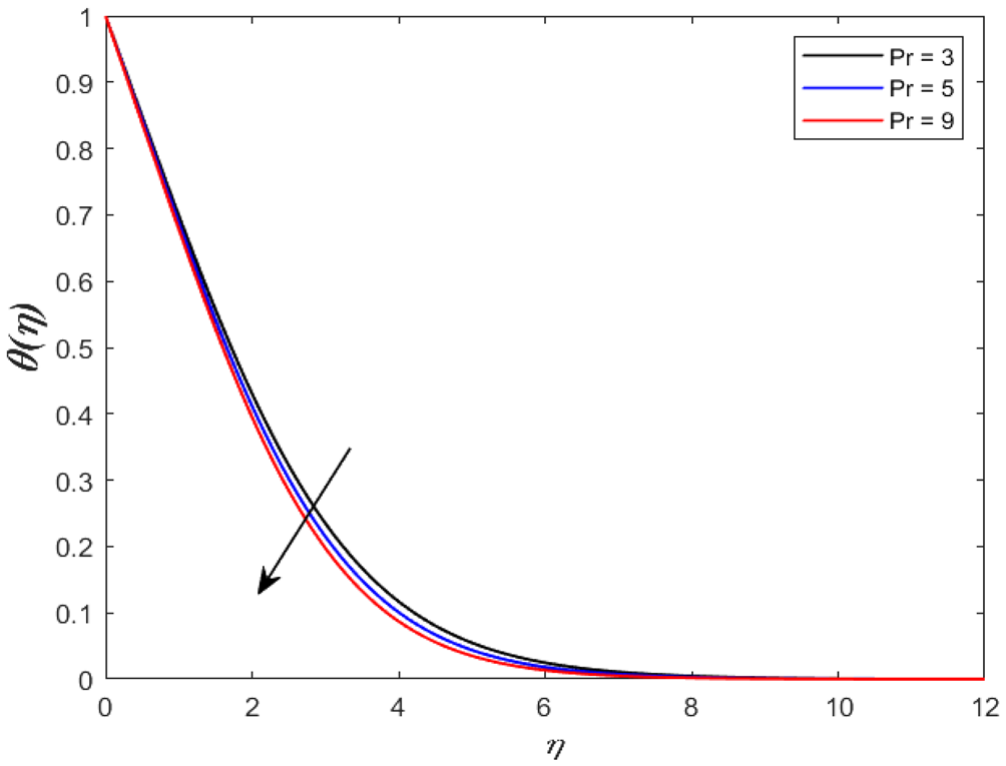
Impact of *Pr* on temperature outline.

**Figure 6:**
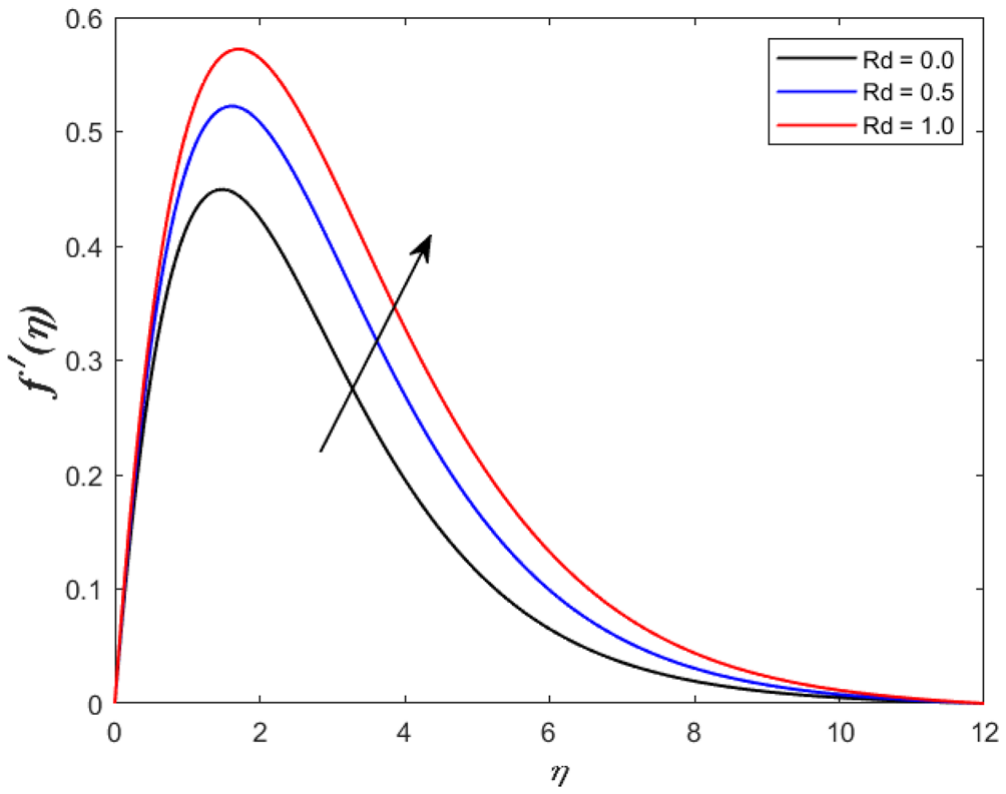
Effect of *Rd* on the velocity outline.

**Figure 7:**
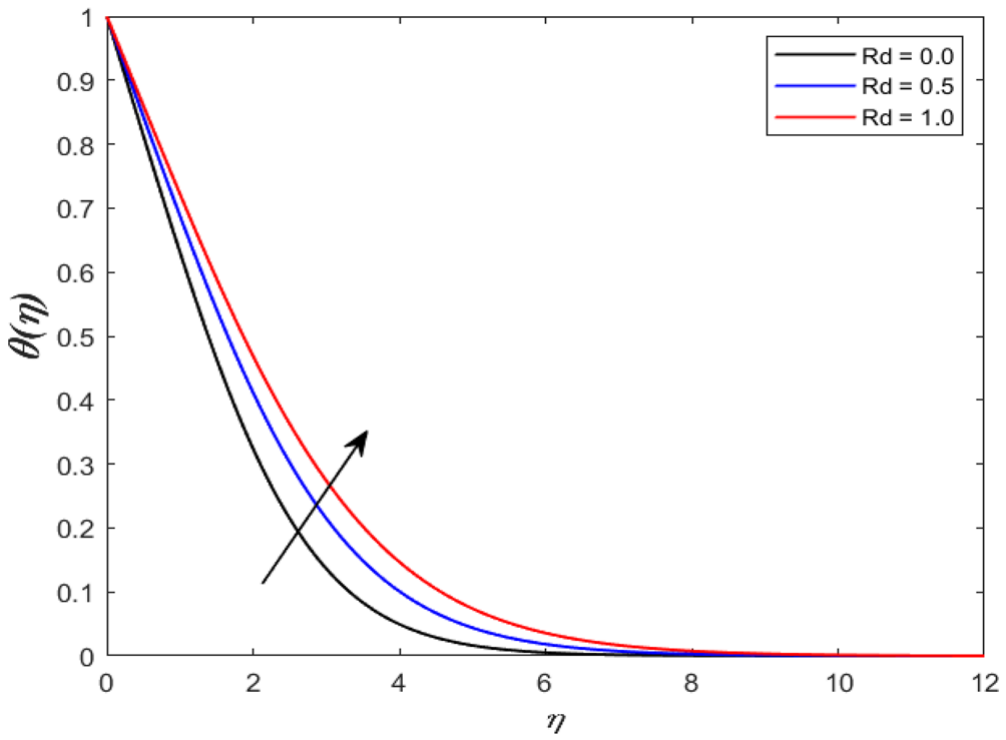
Influence of *Rd* on temperature outline.

**Figure 8:**
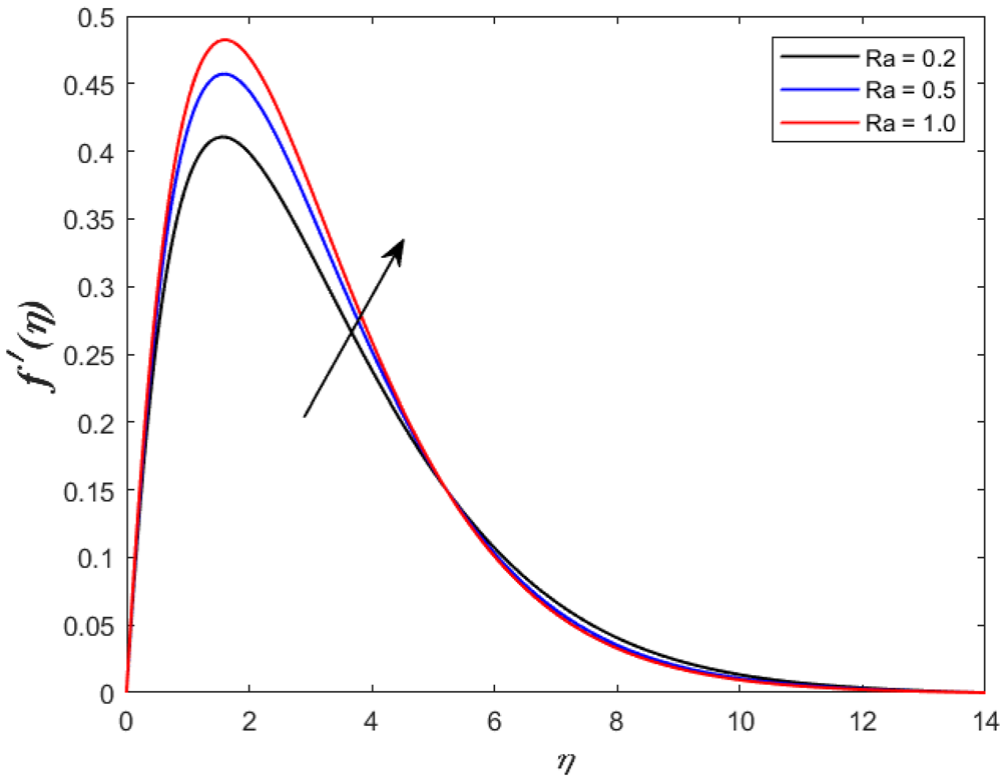
Inspiration of *Ra* on velocity outline.

**Figure 9:**
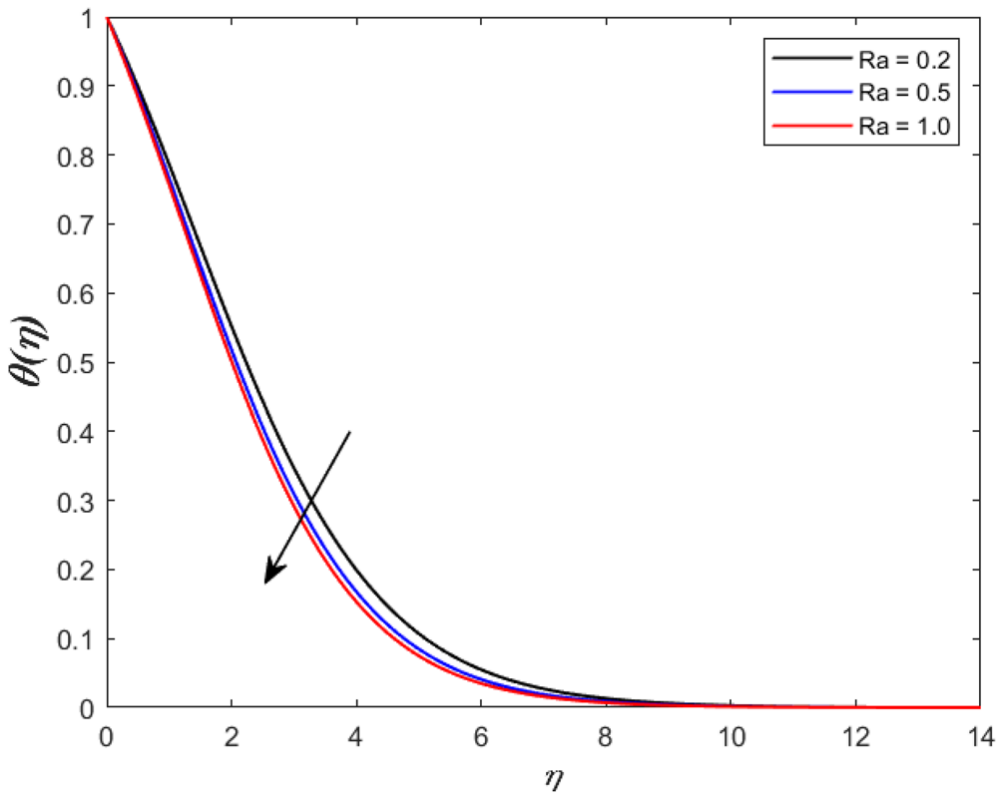
Inspiration of *Ra* on temperature outline.

**Figure 10:**
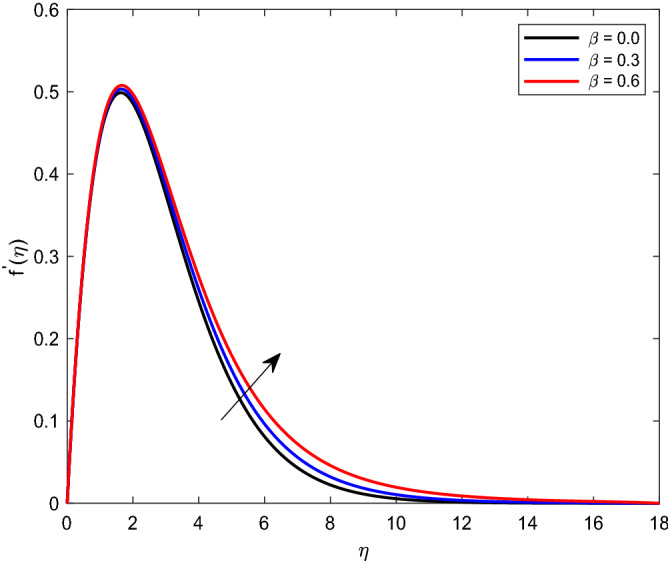
Effect of* β* on the velocity profile.

**Figure 11:**
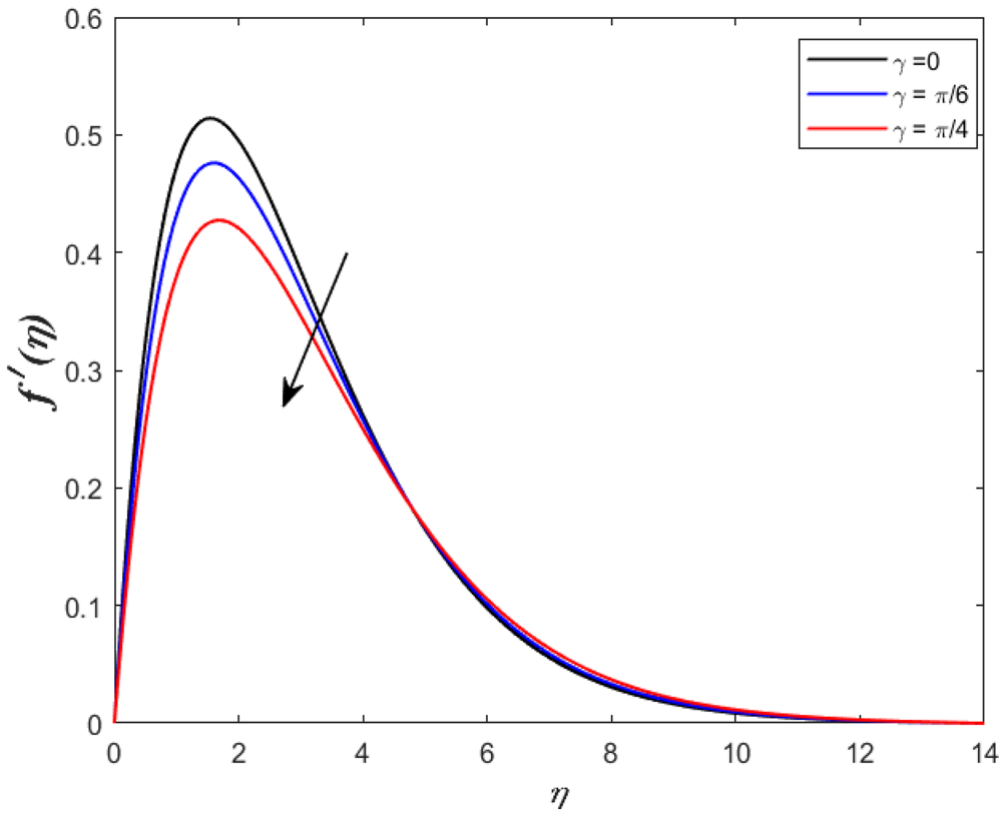
Inspiration of* γ* on velocity outline.

**Figure 12:**
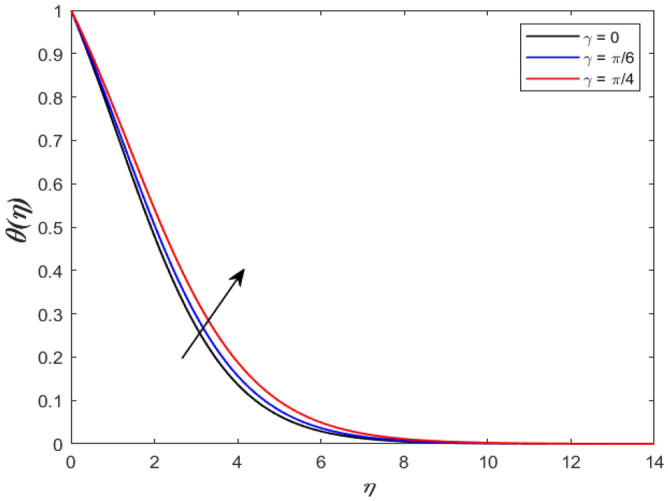
Inspiration γ on temperature field.

**Figure 13:**
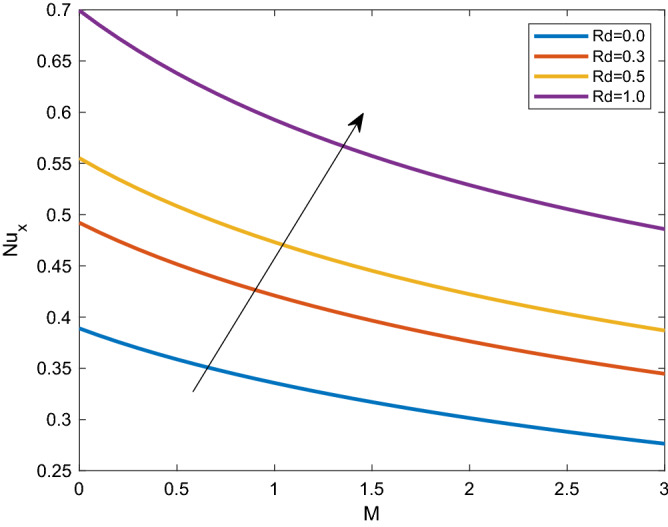
Effect of *Rd* on heat transfer.

A set of graphical figures is presented and discussed here to provide a clear physical understanding. The results are plotted as velocity profiles, temperature distributions, and local Nusselt numbers for a wide range of governing parameters. The effect of *M* on the velocity profile is shown in Fig. 2. It shows that the velocity field decreases as *M* increases. This is because the strong magnetic field inside the boundary layer raises the Lorentz force, which strongly opposes the flow in the opposite direction. As a result, higher magnetic parameter values cause the fluid velocity to decrease. It is also worth noting that the existence of a magnetic field reduces velocity near the wall. Because the magnetic force of the high magnetic field resists fluid movement. The graph indicates that the velocity field decrease and the velocity boundary layer thins as *M* grows, indicating that the magnetic parameter *M* restricts Fluid flow. The Lorentz force grows when the parameter M is increased. This force causes the Fluid flow on the boundary layer to slow down. Figure 3 shows the impact of *M* on the temperature profile. Increasing magnetic field values will increase the temperature field, which will maximize the depth of the energy boundary. The temperature boundary layer profile is enriched with rising values of magnetic parameter *M*. This is due to the Lorentz force caused by the magnetic field, which heated up the Fluid. It is also noticed that the magnetic parameter slows velocity while increasing temperature profiles across the domain in Fig. 4. Velocity grows as the Prandtl number *Pr* does. The Prandtl number is a critical metric in many engineering and industrial systems. Choosing an adequate Prandtl number increased production quality. Figure 5 illustrates the effect of changing *Pr* values on temperature. It shows that the temperature profile decreases for increasing the value of Pr. Due to the size of the Prandtl number relating to the ratio of momentum to heat diffusivity, it is shown that *Pr* significantly affects the depth of the temperature boundary layer and that the smaller the *Pr*, the thicker the temperature boundary layer. As a result, as the *Pr* rises, the temperature lowers and the heat diffusion capacity declines. These results demonstrate the importance of *Pr* in convection flow and heat transfer. Figure 6 shows the effect of *Rd* on the velocity profile. It clearly shows that the velocity profile also does as the *Rd* increases. This is a reasonable observation because the increase in radiation shows that heat energy is moving. Figure 7 displays the impact of *Rd* on the temperature profile. It indicates that the temperature profile also increases when *the Rd* value rises. This is due to the radiation parameter *Rd* transferring extra heat to the Fluid, raising its temperature, and the velocity profile grows as *Rd* increases. The thermal boundary layer converges quicker with the parameter than the momentum boundary layer. This is because injecting thermal radiation into the Fluid adds more heat, increasing the temperature and thickness of the Fluid’s boundary layer. Figures 8 and 9 illustrated the impact on velocity and temperature profile. It clearly shows that the velocity field was raised for the increasing value of *Ra*, while the temperature profile was lowered to increase the values of *Ra*. This is because the Rayleigh number states the relation between *Gr* and the Prandtl number. Rayleigh number also helps to find the type of fluid flow. The relationship between the velocity profile and *β* can be observed in the Fig. 10. Velocity can be raised by raising the value of *β*. Physically, the rise is caused by a growth in the Fluid’s viscosity and the average absorption coefficient, which increases velocity. The consequences of the relaxation time parameter on the rate are a rise in velocity. The influence of the γ on the velocity and temperature field is exposed in Figs. 11 and 12. We noticed that the temperature profile changes in the same direction as the inclination angle grows, decreasing velocity. With the help of this figure, the impacts of the plate’s inclination angle on the temperature profile are investigated. It is discovered that increasing the inclination angle causes a rise in the thermal buoyancy effect, resulting in a decreased velocity field and increased maximum temperature. The impacts of the *Rd* on the *Nu*_*x*_ are exposed in the Fig. 13. When *Rd* is more significant, the local Nusselt number rises. This is because the momentum boundary layer thickens with growing radiation parameter values. As the radiation parameter is increased, so are the values of the Nusselt number. This graphic presents information on the flow’s heat transfer properties in an easy-to-use manner for study and engineering calculations. This illustrates that the *Rd* significantly impacts the heating and cooling processes.

Table 2 represents *C*_*f*_ variations for different parameters *Pr*, β, *Ra*, *M*, *k*_1_, *γ*, *Rd*, Q*. It shows that *C*_*f*_ declines for the increasing values of the parameters β, *Ra*, *M*, *k*_1_, *γ*, but *C*_*f*_ increases for the rising importance of *Rd*, *Pr*, Q*.

**Table 2:** Skin friction variation for various parameters values.

*Pr*	Β	*Ra*	*M*	*k*_1_	*γ*	*Rd*	Q*	*Cf*
5	0.1	0.5	0	0.5	30^0^	0.5	0.01	0.858972
5	0.1	0.5	1	0.5	30^0^	0.5	0.01	0.695408
5	0.1	0.5	2	0.5	30^0^	0.5	0.01	0.601602
5	0.1	0.5	3	0.5	30^0^	0.5	0.01	0.537811
5	0.1	0.5	0.5	0.5	30^0^	0	0.01	0.704145
5	0.1	0.5	0.5	0.5	30^0^	0.3	0.01	0.743108
5	0.1	0.5	0.5	0.5	30^0^	0.5	0.01	0.763252
5	0.1	0.5	0.5	0.5	30^0^	1	0.01	0.801806
1	0.1	0.5	0.5	0.5	30^0^	0.5	0.01	0.659184
3	0.1	0.5	0.5	0.5	30^0^	0.5	0.01	0.734663
5	0.1	0.5	0.5	0.5	30^0^	0.5	0.01	0.763252
9	0.1	0.5	0.5	0.5	30^0^	0.5	0.01	0.790960
5	0.1	0.1	0.5	0.5	30^0^	0.5	0.01	0.819191
5	0.1	0.3	0.5	0.5	30^0^	0.5	0.01	0.789581
5	0.1	0.5	0.5	0.5	30^0^	0.5	0.01	0.763252
5	0.1	0.7	0.5	0.5	30^0^	0.5	0.01	0.739573
5	0.1	0.5	0.5	0.1	30^0^	0.5	0.01	0.819191
5	0.1	0.5	0.5	0.3	30^0^	0.5	0.01	0.789581
5	0.1	0.5	0.5	0.5	30^0^	0.5	0.01	0.763252
5	0.1	0.5	0.5	0.7	30^0^	0.5	0.01	0.739573
5	0.1	0.5	0.5	0.5	0^0^	0.5	0.01	0.860877
5	0.1	0.5	0.5	0.5	30^0^	0.5	0.01	0.763252
5	0.1	0.5	0.5	0.5	45^0^	0.5	0.01	0.643627
5	0.1	0.5	0.5	0.5	60^0^	0.5	0.01	0.479813
5	0.1	0.5	0.5	0.5	30^0^	0.5	0.01	0.763252
5	0.3	0.5	0.5	0.5	30^0^	0.5	0.01	0.764903
5	0.5	0.5	0.5	0.5	30^0^	0.5	0.01	0.766575
5	0.7	0.5	0.5	0.5	30^0^	0.5	0.01	0.768267
5	0.1	0.5	0.5	0.5	30^0^	0.5	− 0.1	0.724005
5	0.1	0.5	0.5	0.5	30^0^	0.5	0.0	0.759451
5	0.1	0.5	0.5	0.5	30^0^	0.5	0.01	0.763252
5	0.1	0.5	0.5	0.5	30^0^	0.5	0.03	0.771002

Calculated values of *Nu*_*x*_ for various values of *M* = 0.5, *Rd* = 0.5, *Pr* = 5, *Ra* = 0.5, *γ* = *pi*/6*. β* = 0.1, Q* = 0.01. The local Nusselt number values in Table 3 represent the increasing values of the *Rd* and heat generation parameters. We can infer from this the Nusselt number increased and diminished as the values of the parameters *Rd* and Q* increased.

**Table 3:** Local nusselt number for various values.

*Rd*	Q*	*Nu*_*X*_
0	0.01	0.358637
0.3	0.01	0.451205
0.5	0.01	0.507495
1	0.01	0.634964
0.5	− 0.1	0.629836
0.5	0.0	0.519172
0.5	0.01	0.507495
0.5	0.03	0.483781

## Conclusion

This study quantitatively investigated steady-state free convection and heat transfer over an inclined plate on a heat-producing, thermal radiation, and porous medium in the presence of MHD. The investigation shows velocity, temperature and the Nusselt number profile on dimensionless parameters, namely *Pr*, *M*, *Rd*, inclined angle parameter, *Ra* and Maxwell Fluid parameter. The resulting specific conclusions are obtained using Bvp4c in MATLAB. An analysis is done on the inspiration of *Q* and *Rd* on steady MHD flow on an inclined perpendicular plate submerged in a porous material. Following are the findings of this research. This flow has been used in processes involving higher temperatures and space technologies; for growing values of the parameters *Pr*, *Rd*, and *Ra*, the velocity outline increases, but for increasing values of *M*, the velocity field reduces. And it is determined that the temperature field is grown for the parameter *M*, *Rd*, whereas the temperature outline is decreased for the growing parameter *Pr*, *Ra*. The conclusion was that the local Nusselt number grows as *Rd* increases in value.

## Data Availability

The datasets used during the current study available from the corresponding author on reasonable request.

## List of symbols

*λ*: Relaxation time
*q*_*r*_: Radiative heat flux
*f*: Dimensionless stream function
*T*_*w*_: Temperature on the wall
*c*_*p*_: Specific heat
*T*: Fluid temperature
*C*_*f*_: Skin friction coefficient
*σ*: Fluid’s electric conductivity
υ: Kinematic viscosity
*k*_1_: Porous medium
*k*: Fluid’s thermal conductivity
*g*: Gravitational acceleration
*η*: Similarity variable
*Nu*: Heat transfer coefficient
*ρc*_*p*_: Heat capacity
*M*: Magnetic parameter
*ρ*: Fluid density of the particles
*ψ*: Non-dimensional stream function
*θ*: Non-dimensional temperature
*θ****: Dimensionless temperature
*σ**: Stefan–Boltzmann constant
*μ*: Dynamic viscosity
